# A Multifunctional Mineral Binder for Plywood Production: The Effect of Manufacturing Parameters on Bonding Quality

**DOI:** 10.3390/ma13102360

**Published:** 2020-05-21

**Authors:** Ali Shalbafan, Amin Nadali, Heiko Thoemen

**Affiliations:** 1 Department of Wood and Paper Science and Technology, Faculty of Natural Resources and Marine Sciences, Tarbiat Modares University, Noor, P.O. Box 46414-356, Iran; amin.nadali@modares.ac.ir; 2 Architecture, Wood and Civil Engineering, Bern University of Applied Sciences, CH-2500 Biel 6, Switzerland; heiko.thoemen@bfh.ch

**Keywords:** mineral binder, geopolymer, plywood, formaldehyde emission, lay-up time, veneer roughness, veneer layouts, compression ratio, press temperature

## Abstract

Geopolymers show great potential for use as binders in developing and manufacturing multifunctional wood products. The objective of this study was to improve the bonding quality of a geopolymer binder, with wood veneers, using different manufacturing parameters. To this end, we produced five layered plywood panels treated with various lay-up times (1, 5, 10, 15 min), panel compressibility values during hot pressing (5%, 10%, 15%, and 30% compression), veneer roughness values (low, medium, and high roughness), press temperatures (120, 140, and 160 °C), and veneer layouts via changing the middle layer position of plywood relative to the surface layers. The results show that the shear strength and thickness swelling were negatively influenced by increasing the lay-up time of resinated veneers and panel compressibility. Increasing the veneer roughness significantly increased the panels’ properties. Furthermore, the panels produced with a pressing temperature of 140 °C showed the best performances. The veneer layouts also significantly changed the physical and mechanical properties of the plywood panels. Generally speaking, the results obtained in this study show that improving the bonding quality of geopolymer binders with wood can be done through the manipulation of plywood manufacturing parameters.

## 1. Introduction

Multifunctional (wood) products offer a practical solution for improving energy and material efficiencies. The adhesives used in wood-based products (WBPs) play a critical role in developing multifunctional wood products [1,2]. Adhesives require new and improved materials and manufacturing processes, as well as innovative actions to increase their efficiency and reliability to achieve multifunctional wood products [1]. Fire resistance, antifungal properties, UV-stability, and health-related factors (e.g., formaldehyde emissions) are the properties that should be most strongly considered for novel wood products. The production of WBPs currently almost fully depends on the usage of synthetic binders, especially formaldehyde-based binders, due to their low costs and excellent properties [2,3,4]. Formaldehyde has been classified as a human carcinogen substance, thereby restricting its further usage in building applications, especially in the USA and Europe. Much effort has been made by researchers to minimize or eliminate formaldehyde emissions through various strategies. Some of the techniques performed to minimize formaldehyde emissions, include optimization of the formaldehyde to urea molar ratio, the modification and condensation of resin, production process optimization, and the utilization of formaldehyde scavengers [1,5,6]. The following are some of the challenges related to these strategies: Short-term effects on emission reductions, negative effects on panel properties, higher costs, and challenges related to production processes. However, the development of formaldehyde-free binders is another important research area that offers several applications [7,8]. In this context, mineral (inorganic) binders can address the aforementioned challenges via a rational combination and synthesis of mineral admixtures with the unique structural properties of wood. Mineral binders include formaldehyde-free properties, as well as high resistance to fire, decay, and UV-stability [9,10,11]. To develop such binders, a profound understanding of manufacturing parameters, and detailed information on material synthesis and characterization, are essential [12,13].

Gypsum, ceramic, and cement (Magnesia and Portland) are some of the traditional mineral components that are extensively used for manufacturing wood–mineral composites. Recently, both Portland cement and magnesia-based components were used as binders to produce laboratory plywood panels [10,14]. However, the use of these mineral binders in WBPs has some limitations in terms of the environmental impacts caused by the extraction of raw materials, carbon dioxide emissions, and technological challenges during manufacture [9,15]. To address these concerns, another form of cementitious materials called a geopolymer has gained greater interest in recent years [16,17]. Geopolymer binders, as an emerging class of mineral materials with multifunctional characteristics, can be manufactured from aluminosilicate powder (with high amounts of silica (SiO_2_) and alumina (Al_2_O_3_)) activated by an alkaline solution/activator [18]. Besides not emitting formaldehyde, fire resistance, antifungal properties, UV-stability, and much lower greenhouse gas emissions are other interesting features of geopolymer binders that can fulfil the market’s need for the production of novel multifunctional products [16,19,20].

Some publications used of wood/lignocelluloses materials as reinforcement elements in geopolymer compositions [9,15,21]. The binding ability between wood and a geopolymer, provided by the physical interaction between the molecules, was shown to be sufficient in dry conditions [11]. Other studies also showed that the workability of a geopolymer binder with wood is highly dependent on the ratios of the materials used for binder formulation [12,13,22]. The potential of using a geopolymer binder for producing various types of WBPs (e.g., plywood and laminated veneer lumber) has been recently confirmed by researchers [23,24]. It is believed that the further development and possible applications of WBPs, using geopolymer binders, can be accelerated by enhancing the bonding quality between the geopolymer binders and the wood surface.

The current bonding capacity between wood and mineral binders, especially a geopolymer, is insufficient, primarily due to the geopolymer networks’ extreme brittleness and low interfacial compatibility with wood [11,23,25]. In addition, wood inherently shrinks and swells in response to hot and humid conditions, which creates peeling or cracking in the wood’s structure [4,26]. Therefore, the adhesive used to produce wood products should have adequate toughness to tolerate the wood’s dimensional instability [2,24,27]. The surface quality of the adherent (wood) is also important for the improvement of adhesion quality [28,29]. Although the adhesion mechanisms between wood and geopolymer components are not well defined, mechanical interlocking is the most feasible bonding mechanism created between wood and a geopolymer [23,30,31]. Many studies have already been conducted to improve the bonding quality between wood and synthetic binders [28,32,33,34,35,36,37,38], but, to date, no detailed research has been performed on the improvement of the bonding mechanisms between wood and mineral components, especially geopolymers. The bonding quality and interface structure of a binder with wood can be changed via the manipulation of the manufacturing parameters. Hence, exploring the effects of various manufacturing parameters on the bonding quality between a geopolymer binder and wood, as well as the plywood’s properties, were the major objectives in this study. The production parameters used in this study were the lay-up time of the resinated veneers, the compression ratio of the panels, the surface roughness of the veneers, the pressing temperature, and the veneer layouts.

## 2. Materials and Methods 

### 2.1. Materials

Commercially rotary-cut sliced poplar veneers were supplied from the “Afra Veneer” Company in Mazandaran Province, Amol, Iran. Defect-free veneers were prepared with dimensions of 400 × 400 × 1.5 mm^3^ and a moisture content of 9.5%.

Commercial metakaolin (trade name ARGICAL M1000) was purchased from Imerys Fused Minerals GmbH (Laufenburg, Germany) as an aluminosilicate powder. According to technical datasheet, metakaolin has a specific surface area of 17 m^2^/g (determined by Brunauer, Emmett, and Teller) and a pH of 6. The metakaolin was activated by an alkaline solution made of sodium silicate and potassium hydroxide to produce the geopolymer binder. Inorganic sodium silicate (2.4 molar ratio of SiO_2_:Na_2_O) was supplied from Industry Silicate Iran (Qazvin, Iran) with a pH of 12.3, a viscosity of 625 mPas, and water content of 55%. Potassium hydroxide flakes (KOH) were purchased from BASF AG (Ludwigshafen, Germany).

### 2.2. Binder Synthesis

The geopolymer binder is produced by the activation of aluminosilicate powder with an alkaline activator. The efficiency of the alkaline activator depends on certain factors, such as the type of activator, dosage, ambient temperature, and final water content of the activator. In this study, the geopolymer binder was prepared according to the previous study of Shalbafan et al. [12]. In summary, potassium hydroxide and sodium silicate were dissolved in an appropriate amount of water (depending on the final binder’s solid content). The resulting alkaline solution was cooled down to around room temperature due to the exothermic reaction of the dissolution. Finally, the metakaolin powder was gradually added to the prepared alkaline solution and blended with a mixer (1000 rpm) until a homogenous mixture was achieved. Importantly, the molar ratio of SiO_2_:Al_2_O_3_, Na_2_O:SiO_2_ and Na_2_O:Al_2_O_3_ in geopolymer binder were 3.1, 0.11 and 0.33, respectively. The final geopolymer binder’s solid content was set at 70% prior to application.

### 2.3. Plywood Production

Five-ply plywood panels were made under laboratory and conventional conditions using the geopolymer as a binder. The geopolymer binder was spread manually on the veneers’ surfaces with a hand roller to obtain the most uniform binder layer. After binder application, the neighbouring veneers were oriented, layer by layer, forming the plywood panels according to conventional method. The ply stacks were then pressed in a laboratory single opening hot-press (Ranjbar Press Ltd., Isfahan, Iran). For all plywood panels, a similar hot-pressing schedule was performed. The press time were 600 s. The press pressure and temperature, panel thickness, and layer orientations were also controlled according to the panels’ treatments mentioned in the following section.

The spread amount of the geopolymer binder was equal at 420 g/m^2^ on each glue line to achieve full coverage of the veneer faces; this quantity was calculated based on the veneer area and the density of the geopolymer binder (about 1850 kg/m^3^). Three plywood panels were manufactured for each of the treatments, resulting in a total of 42 panels. Prior to any process step, both the veneer sheets and plywood panels were conditioned at 65 ± 3% relative humidity and a 20 ± 2 °C temperature for two weeks. Then, the plywood panels were cut into test samples for characterization.

### 2.4. Panels Variables

The efficiency of the various manufacturing parameters for the bonding quality of the geopolymer and the wood was tested. The treatments used were as follows:

- lay-up time (cold-tack time) of the resinated layers;
- compression ratio of the panels;
- veneers with various surface roughness;
- press temperature;
- veneer layouts (the position of middle layers relative to the surface layers).

A summary of all variables for plywood production is presented in Table 1. Four lay-up times were defined (1, 5, 10, and 15 min) to observe their effect on bonding quality and the plywood properties. The interval between assembly of the last resinated veneer sheet and the start of hot pressing was counted as the lay-up time. The compression ratio of the plywood panels was selected as the second most important parameter influencing the plywood performances. The compression ratio of the plywood panels (CRP) was calculated as follows,

CRP = (T_V_ − T_P_)/T_V_ × 100 (1)

where CRP (%) is the compression ratio of the plywood panels, T_V_ (mm) is sum of the thickness of the veneers prior to panel production, and T_P_ (mm) is the final thickness of the plywood panels after hot pressing (mm). In this section, four different compression ratio were selected (5%, 10%, 15%, and 30%) to produce the plywood panels. The different compression ratios of the plywood panels were achieved using various press pressures. 

**Figure 1:**
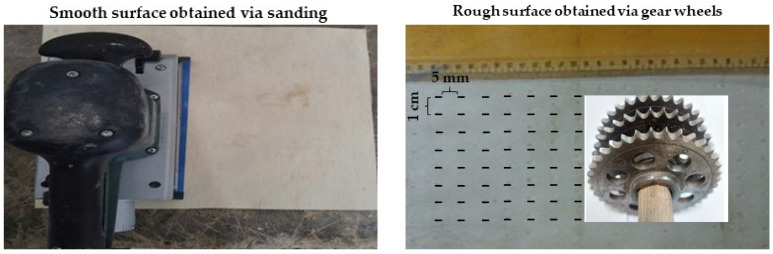
The surfaces of smooth and rough veneer sheets.

Another treatment involved the veneer roughness. Different techniques were performed on the tight side of each veneer to create various types of roughness on the veneer surface. The veneers were sanded with sanding paper No. 400 to give the veneers low surface roughness. In addition, another set of veneers was passed through sophisticated gear wheels that incised the top surface with chisel-shaped holes (5 mm length and 2 mm width in each hole) to create a veneer surface with high roughness. The veneers sheets without any pre-treatments were those with medium roughness. Figure 1 shows the surfaces of the smooth and rough veneer sheets prior to plywood production. 

**Figure 2:**
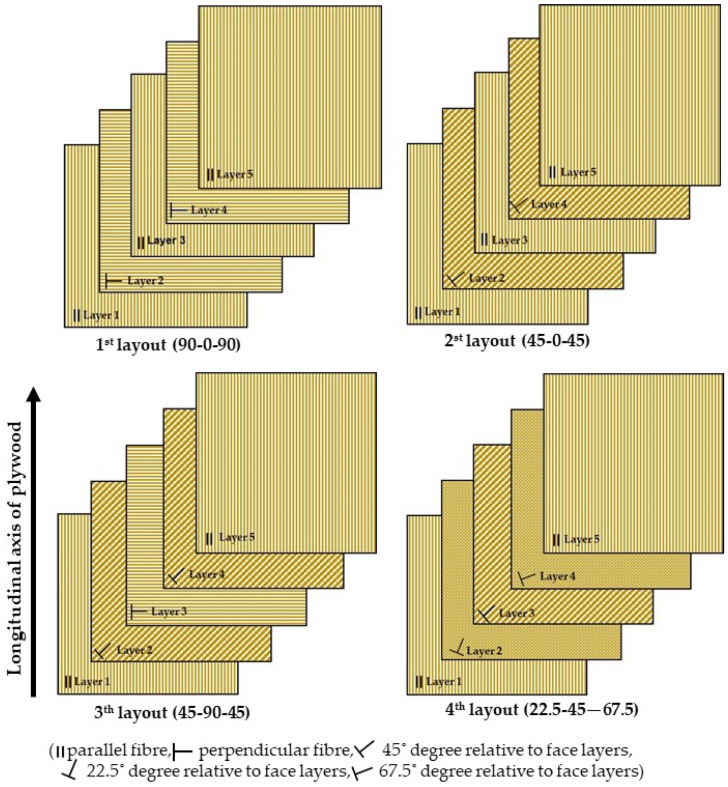
Various types of veneer layouts used for plywood production (numbers in parenthesis are the middle layers fibre angle relative to surface layers).

The hot-pressing temperature influences the bonding quality and plywood properties. Therefore, a temperature range of 120 to 160 °C was used for the hot-pressing of the plywood panels using the geopolymer as binder. Finally, the veneer layout also affects the plywood panels. Hence, the fibre angle (position) of the middle layers, relative to the surface layers of the plywood panels, were varied to create four different plywood layouts. The layers numbers 2, 3, and 4 were called the middle layers for the 5-ply plywood panels. Importantly, for all layouts, the grain direction of the surface layers was constant and parallel to the longitudinal axis of the panel. Figure 2 shows the various types of veneer layouts used for plywood production. 

### 2.5. Plywood Characterisation

The mechanical and physical properties of the plywood panels produced with various treatments were investigated. The shear strength of a plywood sample (120 × 25 mm^2^) was measured by a single lap-shear test according to the EN 314 standard [39] for plywood bonding class 1 (interior conditions) and class 2 (covered exterior conditions). For interior conditions, the samples were immersed in water at 20 ± 2 °C for 24 h. For the covered exterior conditions, the samples were placed in hot-water at a temperature of 98 ± 2 °C for 6 h, removed from the hot-water, and then cooled by water soaking at a temperature of 20 ± 2 °C for at least 1 h prior to the shear strength test. The untreated samples (dry samples) were also tested for comparison. Shear tests were conducted using a Santam universal testing machine (Santam Engineering Design Company, Tehran, Iran) with 0.25 mm/min as the constant cross-head displacement rate. The reported mean of the shear strength represents an average of 15 samples for each plywood structure.

The bending strength (MOR) and modulus of elasticity (MOE) were measured according to the EN 310 [40] on samples with parallel directions to the fibres of the surface layers. Bending tests were performed using a Santam universal testing machine (Santam Engineering Design Company, Tehran, Iran) with a 5.5 mm/min constant cross-head displacement rate. For each plywood structure, nine bending samples were tested.

The short- and long-term thickness swelling was determined after 2, 24, 48, 96, 192, 386, and 768 h of water soaking according to EN 317 standard [41] with the sample size of 50 × 50 mm^2^. The water absorption (WA) of the samples was also calculated according to the following formula,

WA (%) = ((m_t_ − m_I_)/m_I_) × 100 (2)

where WA is the water absorption at time t, m_t_ and m_I_ are the weights of the samples at time t (2, 24, 48, 96, 192, 386, and 768 h) and the weight of the samples prior to water soaking, respectively. Nine replications for each panel variation were tested. Changes in the physical and mechanical properties, between the various treatments were analysed, using a one-way analysis of variance (ANOVA) with the SPSS software (IBM SPSS Statistics 25, Armonk, NY, USA). Duncan test was used to differentiate the significant of average values that is indicated by different letters in each graph. The statistical significance was set to *p* < 0.05.

### 2.6. ATR-FTIR Spectroscopy

The effect of different press temperatures on the cured geopolymer binder was characterized by attenuated total reflection-Fourier transform infrared spectroscopy (ATR-FTIR). To this end, the geopolymer binder was taken from the first binder line of the fractured shear specimens (untreated samples), and then was fully milled. A spectrogram of these samples was obtained using a Perkin Elmer spectrometer (Waltham, MA, USA). The spectra were recorded in the range of 4000–550 cm^−1^ at a 4 cm^−1^ resolution.

## 3. Results and Discussion

### 3.1. Lay-Up Time

**Figure 3:**
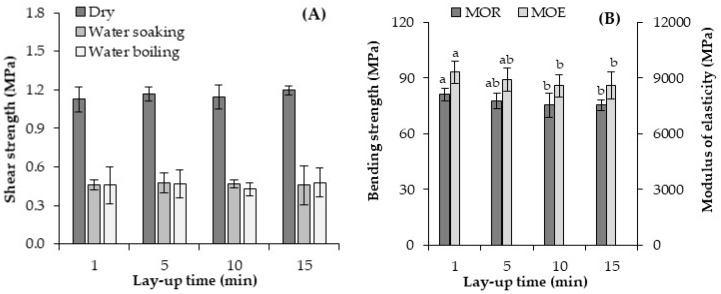
Shear strength (**A**) and bending properties (**B**) of the geopolymer-bonded plywood with various lay-up times. The statistical differences are denoted with various letters in figure columns.

The shear strength of the geopolymer-bonded plywood with various lay-up times is shown in Figure 3A. The shear strength values were not significantly changed when the lay-up time increased from 1 to 15 min. The ability of the binder to develop a cold tack is needed in plywood production, especially during pre-pressing to ensure that the veneers slightly stick together, and can be transported and placed into the hot press for binder curing [32]. Cold tack can be influenced by different factors, such as veneers temperature, veneer moisture content, as well as the lay-up time. Notably, the geopolymer binder features were not negatively affected by increasing the layup time. In other words, the lay-up time presented a minor impact on the shear strength of geopolymer binder. Generally, the highest values of bonding shear strength were observed in the untreated (dry) samples. The shear strength for the untreated and pre-treated samples with various lay-up times was around 1.14 MPa, and 0.46 MPa, respectively. There were no significant changes between the samples immersed in cold (24 h) and boiled water (6 h). With reference to Figure 3, the samples experienced an extreme change in condition after both the water soaking and boiling pre-treatments, which possibility caused more moisture to penetrate the binder line and decrease the shear strength of the plywood samples. The areas exposed to moisture influenced the binder features, deteriorated the quality of the geopolymer bonding, and consequently, reduced the quality of bonding [24]. In other words, the dimensional stability (swelling and shrinkage) of the wood caused the formation of cracks in the wood-binder interface and a decrease in the binder shear strength [11]. Furthermore, water may also cause cracking in geopolymer due to capillary forces.

The average values of the bending strength (MOR) and modulus of elasticity (MOE) in the bending of the plywood samples produced with various lay-up times are presented in Figure 3B. The lay-up times clearly affected the bending properties of the plywood samples. Referring to Figure 3, the plywood made of a geopolymer binder with a one minute lay-up time had an MOR and MOE of about 81 MPa, and 9300 MPa, respectively. A small decrease in the MOR and MOE of the plywood panels was seen when the lay-up time increased to 15 min. Thus, the lowest value of both MOR and MOE was observed for the panels produced with longer lay-up times (15 min), about 75 MPa, and 8600 MPa, respectively. The adhesion manner of the binder applied to the wooden surfaces is a key factor for the development of panels’ properties [4]. It seems that the adhesion quality of geopolymer binder with wooden surfaces were negatively influenced with increasing the lay-up times from 1 to 15 min. Water content of binder plays an important role in the binder penetration into the wooden cells. As the water content of the adhesive decreases, the viscosity of the adhesive is higher and penetration decreases [2]. The water content of the geopolymer binder is decreased by increasing the lay-up times during plywood production, as the wood absorbs part of the mixing water [14]. Therefore, the negative effects, observed for the lay-up time, can be explained by the importance of the binder drying process during plywood production.

In summary, the lay-up times showed no effect on the shear strength of the plywood samples and also negatively influenced the bending properties of the plywood panels. Hence, a lay-up time of one minute was selected for the following variables in the current study.

**Figure 4:**
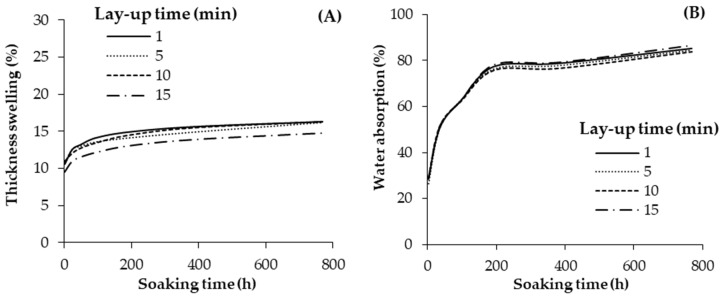
Thickness swelling (**A**) and water absorption (**B**) of the geopolymer-bonded plywood with various lay-up times.

The thickness swelling (TS) and water absorption (WA) of the geopolymer-bonded plywood with various lay-up times are shown in Figure 4. The results show that the various lay-up times had no significant influence on the TS and WA of the plywood samples even after 768 h of water soaking. Although the lowest TS was observed in the panels with a 15 min lay-up time, the difference in this TS compared to the highest TS is only around 1%. Notably, the TS values of the plywood samples did not show a further increase after nearly 100 h of water soaking, indicating that the wood substance in the panels was nearly saturated. Three distinct regimes can be observed for the WA values. A first increasing trend up to around 48 h of water soaking which tended somehow to be leveled off till 96 h. This is possibly due to the filling of voids in the wood cells. A second increase of WA was also observed from 96 h till 196 h of water soaking which tended to be leveled off until 384 h of water soaking. Finally, a steady increase of WA was observed after 384 h of water soaking. The increasing trend of regimes 2 and 3 are possibly due to the additional micro-cracks created in the hardened geopolymer when it was immersed in water [11,24]. As mentioned earlier, water may also cause cracking in geopolymer due to capillary forces. It is noteworthy that none of the plywood samples were delaminated even after 768 h water soaking, showing the better stability of geopolymer-bonded products in water, compared to other organic binders (e.g., adhesives based on tannin, soya, and starch), which suffer from hydrolysis during water immersion [1,2,5,23].

### 3.2. Compression Ratio of Panels

**Figure 5:**
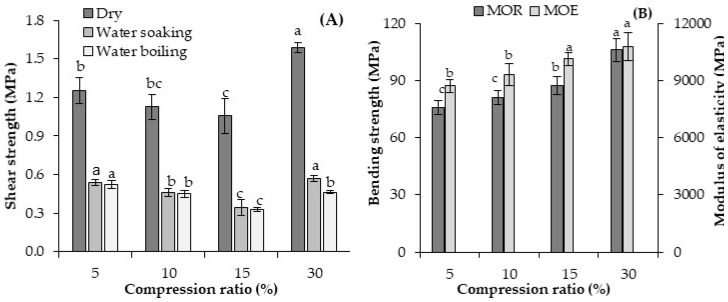
Shear strength (**A**) and bending properties (**B**) of the geopolymer-bonded plywood with various layer compression ratios. The statistical differences are denoted with various letters in figure columns.

The experimentally made geopolymer-bonded plywood, with various compression ratios, was analysed using the shear strength and three point bending tests, and the results are presented in Figure 5. In reference to Figure 5, by increasing the compression ratio from 5% to 15%, the shear strength in the treated samples (water soaking and boiling) decreased by about 37%. This may be explained by the fact that increasing the panels’ compression ratio, resulted in more binders pressed into the vessels and cracks of the veneers, but less remained in the glue line, thereby reducing the bonding strength. These results are consistent with the results of Chang et al. [8] and Bekhta et al. [7] who used plastic sheets as a binder for plywood. Different press pressures were used to reach various panel compression ratios. The compression ratio plays an important role in the plywood’s features, as it is responsible for providing adequate contact between both materials (veneers and binder) and helping the binder flow into the voids and irregularities of the wood veneer [7,42]. Hence, a high compression ratio was recommended for improving the adhesion features of the veneers [8]. However, the obtained results are somewhat contradictory to the results obtained earlier for synthetic adhesives [43]. Unlike synthetic adhesives, such as urea and phenol formaldehyde, the use of which requires slightly high pressing pressure, the application of a geopolymer binder allows the bonding of plywood samples at a significantly lower pressing pressure (i.e., a lower compression ratio). Notably, the shear strength of the samples with a 30% compression ratio increased, returning to the same level of a 5% compression ratio. In other words, the difference in the shear strength values for compression ratios of 5% and 30% is insignificant. This phenomenon can be attributed to the fact that, with a much higher increase in pressing pressure, a slight ejection of the binder from the vessels and cracks occurs, and is maintained in the binder line to improve the bonding quality [7].

The effect of the panels’ compression ratios on the bending properties of the geopolymer-bonded plywood samples shows that both, MOR and MOE were significantly increased by increasing the compression ratio. The lowest MOR and MOE were recorded for the panels with a 5% compression ratio (about 76 MPa, and 8700 MPa, respectively). The MOR and MOE increased by about 40% and 25% by raising the panel compression ratio from 5% to 30%, reaching to about 106 MPa, and 10,800 MPa, respectively. The bending properties of wood-based panels is highly dependent on the panel density. The higher the panel density, the higher the bending properties [26]. The density of plywood samples increased by raising the compression ratio of the panels from 5% to 30%, which increased the bending properties.

Briefly, the compression ratio did not positively influence the shear strength of the geopolymer-bonded plywood. Although the bending strengths of the samples were improved by increasing the panel compression ratio, the panel density was also unfavourably increased. Therefore, from economical and technical points of view, it is more appropriate to produce plywood panels with a compression ratio of 5%.

**Figure 6:**
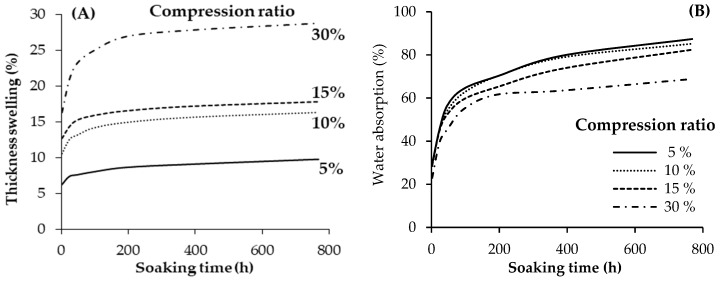
Thickness swelling (**A**) and water absorption (**B**) of the geopolymer-bonded plywood with various compression ratios.

The effect of the panel’s compression ratio on the TS and WA of the geopolymer-bonded plywood after up to 768 h of water soaking is shown in Figure 6. The highest TS was observed in panels with a 30% compression ratio, followed by panels with a 15% and 10% compression ratio. The lowest TS belongs to the panels produced with the lowest compression ratio (5%). In other words, the higher the compression ratio, the higher the TS values of the plywood samples. The TS in wood-based panels was caused by a combination of several factors that are associated with material and manufacturing process variables [26]. Besides the swelling of cellulosic fibres, one of the most important factors often correlated with TS is board density. The boards with a higher density have a more compressive set and a larger spring back than those of a lower density [44]. As mentioned earlier, the density of the plywood panels increased by raising the compression ratio to 30% to create more spring back in the samples during water soaking. The TS of the plywood samples was almost constant at around 100 h of water soaking for all panel treatments.

Referring to Figure 6, the WA values of the plywood samples significantly decreased by increasing the panel compression ratio. The higher the compression ratio of the plywood samples, the lower the WA that was achieved. Higher density boards are characterized by lower voids inside the panels and, therefore, are expected to absorb less water than lower density boards [4,45]. Water can hardly penetrate into the wood’s cell lumen when the board density is higher.

### 3.3. Veneer Roughness

**Figure 7:**
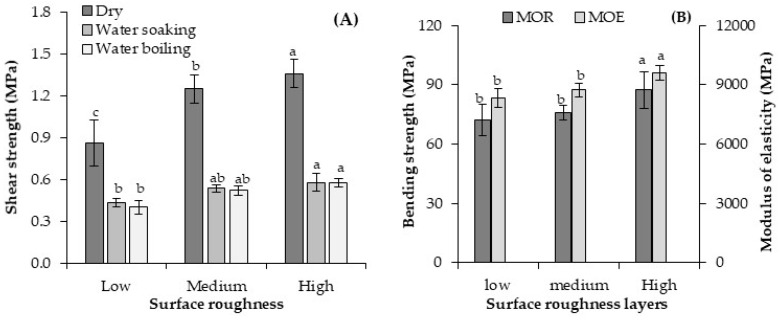
The shear strength (**A**) and bending properties (**B**) of the geopolymer-bonded plywood with various veneer roughness. The statistical differences are denoted with various letters in figure columns.

The shear strength of the geopolymer-bonded plywood with various veneer roughness is presented in Figure 7. Shear strength was the lowest in the plywood samples made of sanded layers possessing a lower surface roughness. The shear strength of plywood samples significantly increased as the veneer roughness also increased. The shear strength of the plywood samples after water soaking and water boiling was recorded at around 0.58 MPa. Indeed, a nearly 40% increase in shear strength was observed in the plywood samples produced with rough surfaces using gear wheels compared to those with smooth veneers. Researchers also showed that plywood produced by incised veneers has a higher bonding quality than plywood made with non-incised veneers [34,37]. Liquid penetration increased by around 24–50% in the inner plies of the incised veneers and created better bonding quality [46]. In other words, increasing surface roughness through various incising processes can improve the entanglement of the veneers with the binder, which enhances plywood bonding [35]. Several levels of adhesive penetration into the wood is feasible via process-induced cracks, cell walls, cell lumens, and pits [27]. Notably, the paths with lower resistance are the most feasible directions for adhesive penetration (such as cell lumens and macro-cracks), although cell wall penetration was introduced as the most vital key to developing strong bonds [29]. While the content of large-scale penetration into veneers is not easy to detect, the incision slits possibly produce process-induced cracks. Here, an increased surface roughness (via incision slits) improved the entanglement of the veneers with the binder and consequently enhanced the plywood bonding.

Referring to Figure 7B, the geopolymer-bonded plywood with smooth veneers showed a MOR and MOE of about 72 MPa, and 8300 MPa, respectively. A significant improvement in both MOR and MOE (parallel to grain) was observed in the plywood made of veneers with higher roughness. The highest MOR and MOE values belonged to plywood samples produced with rough veneers using gear wheels, and were about 87 MPa, and 9600 MPa, respectively. Kymäläinen et al. [34] noted that the incision of veneers improved the modulus of elasticity and bending strengths of birch plywood. Greater mechanical entanglement is possibly created between the veneer surfaces of geopolymer-bonded plywood. This likely results in a better distribution of stress during bending tests and the enhancement of bending properties. This result is also consistent with the trend observed for the shear strength of the plywood samples. Hence, the veneers with higher surface roughness were selected to analyse the following variables of this study.

**Figure 8:**
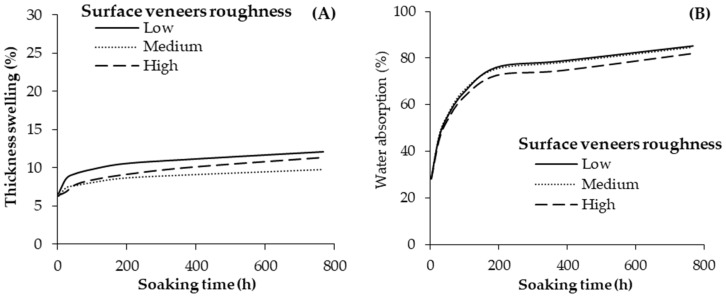
Thickness swelling (**A**) and water absorption (**B**) of the geopolymer-bonded plywood with various veneer roughness.

The average values for the TS and WA of the plywood samples are shown in Figure 8. Increasing the veneer roughness insignificantly affected the TS and WA of the geopolymer-bonded plywood. Referring to Figure 8, TS almost reached a constant level after nearly 100 h of water soaking. The maximum level of the TS was about 11% after 768 h of soaking in plywood produced with smooth veneers. The WA of the samples also increased by increasing the soaking time up to nearly 200 h. A maximum level of nearly 82% water absorption was achieved after 768 h of soaking. In general, increasing the surface roughness of the veneers positively improved the shear and bending properties of the plywood samples, but the TS and WA were not significantly affected.

### 3.4. Hot-Pressing Tempearture

**Figure 9:**
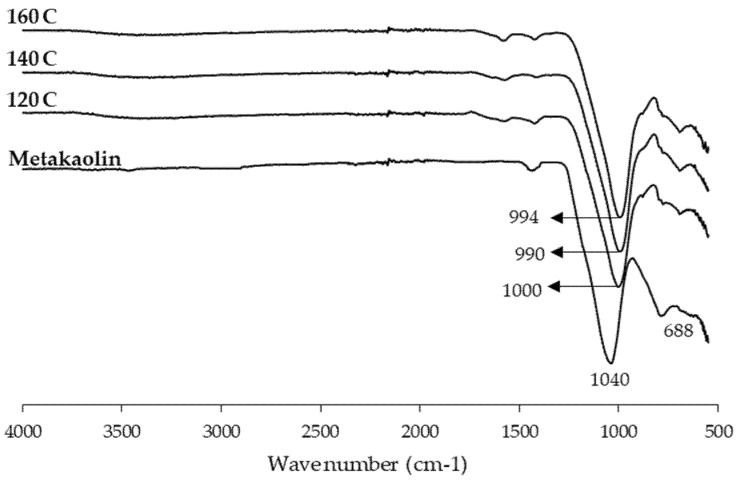
FTIR spectroscopy of the geopolymer binder with various hot-pressing temperatures.

The ATR-FTIR spectra of the metakaolin powder and the cured geopolymer binder at various press temperatures were recorded in the selected spectral range of 550–4000 cm^−1^, and the results are presented in Figure 9. The presence of the large bands at around 1040 cm^−1^ in metakaolin is assigned to the presence of Si-O-Al and Si-O-Si functional groups. Although the relationship between the asymmetric stretching peak positions of various groups of T-O-Si (T = Al and Si) and the extent of the geopolymerisation process are complex, it is still informative to study the geopolymerisation mechanism [16]. The symmetrical stretching of Si-O-Si and Al-O-Si, typical for the formation of an alkaline aluminosilicate network in a metakaolin-based geopolymer, appeared at about 688 cm^−1^ in all binders cured with various press temperatures [18,24]. The stretching vibrations of the Si-O-R bond (R = Na, K, or Si) were observed at 990 cm^−1^ for the geopolymer binder cured at a 140 °C press temperature. This peak in the geopolymer binder with various press temperatures shifted to higher wavenumbers (of about 1000 cm^−1^). Considering the broadness of the Si-O-R^+^ bonds, the higher wavenumbers of this bonds, also in agreement with the literature data [23], can be still related to a weaker geopolymerisation process. The peak at about 1420 cm^-1^ indicates the stretching vibration of the O-C-O band resulting from atmospheric carbonation. The absorption band at about 1570 and 3350 cm^−1^ indicates the bending and stretching vibrations of the O-H groups [16,18,47].

**Figure 10:**
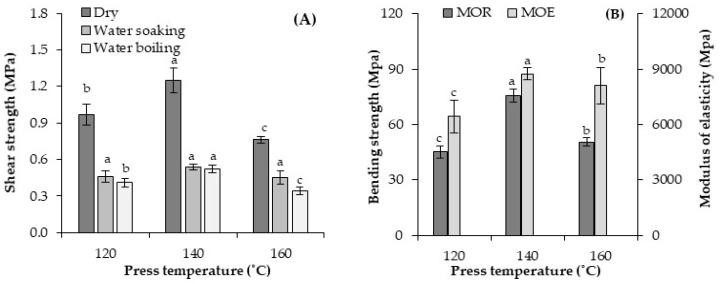
Shear strength (**A**) and bending properties (**B**) of the geopolymer-bonded plywood with various hot-pressing temperatures. The statistical differences are denoted with various letters in figure columns.

The effect of the hot-pressing temperature on the shear strength and bending properties of the geopolymer-bonded plywood samples is shown in Figure 10. In all cases, the press temperature led to a significant change in shear strength. The shear strength of the dry and pretreated samples after water soaking (24 h) and water boiling (6 h) increased by approximately 20% by raising the press temperature from 120 °C to 140 °C. Previous studies also confirmed that a higher press temperature has a positive influence on the shear strength and bonding quality of the geopolymer binder [12,13]. This can be attributed to the higher dissolution of binder ingredients and the successful geopolymer network formation [24]. Notably, the shear strength of the geopolymer-bonded plywood was negatively influenced by further increasing the press temperature from 140 °C to 160 °C. It was shown that the dissolution, polymerization, and reprecipitation processes of the geopolymerization reaction is depended on the curing temperature [47]. The optimum curing temperature of geopolymer binder for plywood production is 140 °C at which the geopolymer samples present the best mechanical properties [24]. A previous study also showed that if higher press temperatures are used for plywood production, a significant reduction in cohesive binder-line strength takes place, resulting in defective plywood [38].

The effect of the hot-pressing temperature on the bending properties of the plywood samples showed the same trend as shear strength. The MOR and MOE were about 45 MPa and 6445 MPa in the panels produced with a press temperature of 120 °C, respectively. Both MOR and MOE reached about 76 MPa and 8730 MPa by increasing the press temperature to 140 °C. Further increasing the press temperature to 160 °C negatively influenced the bending properties. The MOR and MOE were about 51 MPa and 8100 MPa in the panels produced with a press temperature of 160 °C, respectively. As mentioned earlier, a greater crystalline network may be created by the rapid heat development in the geopolymer binder, which may have negatively affected the geopolymer binder features (increasing the binder’s brittleness) [23]. Therefore, a press temperature of 140 °C is preferable for the production of geopolymer-bonded plywood and especially for analyzing the variables in this study.

**Figure 11:**
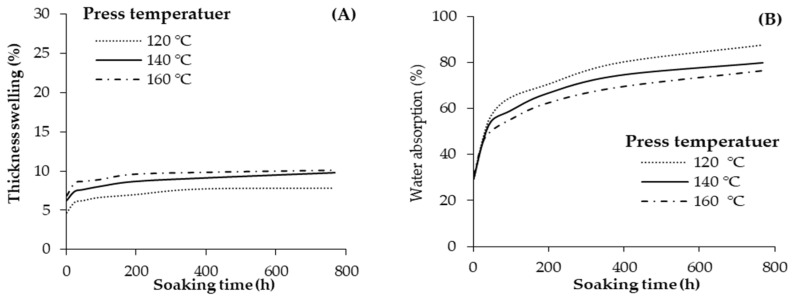
Thickness swelling (**A**) and water absorption (**B**) of the geopolymer-bonded plywood with various hot-pressing temperatures.

The average values of the TS and WA of the plywood samples produced with different pressing temperatures is presented in Figure 11. The lowest and highest thickness swelling values belonged to the samples produced with a press temperature of 120 °C, and 160 °C, respectively. In other words, the TS insignificantly increased with an increase in press temperature from 120 °C to 160 °C. The maximum value of TS was about 10% after 768 h water soaking for the panels produced with a press temperature of 160 °C. Accordingly, the minimum value of TS after 768 h was about 8% for the samples produced with a press temperature of 120 °C. The order of WA values was different with varying the press temperature than that observed for the TS values. The lowest (77%) and highest (87%) WAs after 768 h soaking were observed in the plywood panels produced with press temperatures of 160 °C and 120 °C. The hot-pressing temperature for wood-based composites can influence the functionality of the hydrophilic groups in the woody materials, which contributes to the varying the water absorption and TS of the plywood panels [7,28]. Notably, high press temperature can lead to more softening and more compression of the veneers, which consequently can decrease the WAs [4]. The WA gradually raised with an increase in soaking time from 2 to 768 h. The largest increase in WA occurred during the first 48 h of water soaking.

### 3.5. Veneer Layouts

**Figure 12:**
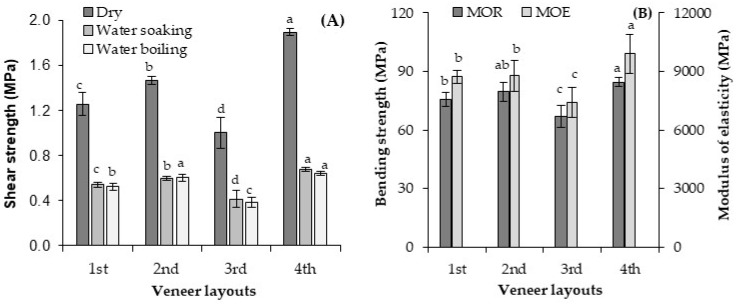
Shear strength (**A**) and bending properties (**B**) of the geopolymer-bonded plywood with various veneer layouts (middle layer angle relative to the surface layers). The statistical differences are denoted with various letters in figure columns.

The influence of veneer layouts on the plywood shear and bending properties was evaluated as the positions (angles) of the middle-layers relative to the surface layers were changed according to Figure 2. The average values of shear strength after various sample treatments (water soaking and boiling) are presented in Figure 12A. The veneer layouts exerted a significant influence on the plywood shear strength. It should be noted that typical plywood has a middle layer position at 90-0-90 degrees relative to the surface layer position (1st layout). Varying the position of the crossover layers from 90 degrees (90-0-90 status) to 45 degrees (2nd layout: 45-0-45 status) relative to the surface layer positively influenced the shear strength of the plywood samples. Here, the shear strength was raised by about 11–17%, depending on the sample treatment, changing the position of the crossover layers from 90 to 45 degrees. The lowest shear strength belongs to the 3rd veneer layout with a middle layer orientation of 45-90-45 degrees. Alternatively, the fourth veneer layout with a middle layer orientation of 22.5-45-67.5 degrees relative to the surface layer attained the highest shear strength among the various veneer layouts. The shear strength in the plywood with the 4th veneer layout had approximately a 23–52% higher shear strength, depending on the sample treatment (water soaking and water boiling), in comparison to the conventional plywood with the 1st veneer layout (90–0-90 degrees) relative to the surface layers. The position and orientation of each veneer in the plywood structure exerted a significant effect on the mechanical properties of the final panels [36,48]. Possibly, the binder’s penetration into the superficial wood cells and its mechanical interlocking with the wood cells changed as the veneer layouts changed. This could influence the shear strength of the plywood samples.

The effect of veneer layout on the bending properties of the plywood samples (Figure 12B) showed a similar trend to shear strength. The lowest bending properties were observed for panels whose 3rd veneer layouts had a 45-90-45 middle layer status relative to the surface layers. Consequently, the highest bending properties were achieved in the plywood samples whose 4th veneer layouts has a 22.5-45-67.5 status. The reason for this seems to be the alignment of the cellulose microfibrils in the veneers, which showed a better effect on the bending stress transfer and the bending properties of the specimens. Apart from the wood species, plywood properties depend on the veneer’s quality, thickness, and number of layers; the veneer layouts; and the adhesive used for plywood bonding [4]. Popovska et al. [36] improved the mechanical properties of plywood samples by varying the panel structures and veneer layouts. It can be concluded that the veneer layouts in plywood structures provide the opportunity to manufacture panels with different strength characteristics. 

**Figure 13:**
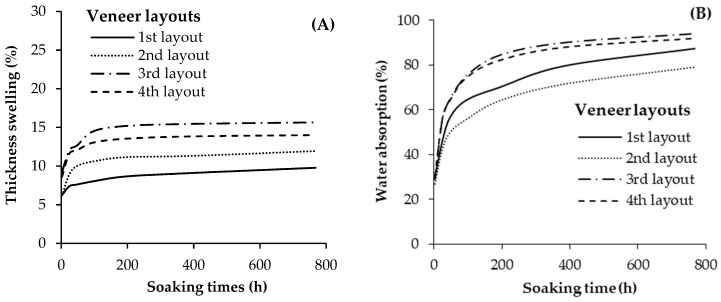
Thickness swelling (**A**) and water absorption (**B**) of the geopolymer-bonded plywood with various middle layer angles relative to the surface layers.

The average values of TS and WA for the geopolymer-bonded plywood samples produced with different veneer layouts are presented in Figure 13. The results showed that varying the veneer layout had a significant influence on the physical properties of the plywood panels. The lowest TS was observed in plywood panels with conventional veneer layouts (First layout: with a middle layer angle of 90-0-90 relative to the surface layers). The highest TS was observed in panels with the 3rd layout featuring a middle layer angle of 45-90-45 degrees relative to the surface layers. The TS reached a nearly constant level after around 72 h of water soaking. It is well-known that the veneer layout has an important influence on controlling the internal stresses created during the swelling of wood and wood products [4,26]. More or less the same trend as TS was observed for the WA values. The lowest and highest water absorption values were observed in the samples produced with the second (45-0-45 degrees), and third (45-90-45 degrees) veneer layouts, respectively. The lowest and highest WA after 768 h of soaking was about 79%, and 94%, respectively. It seems that varying the middle layer’s orientation influenced the penetration and accessibility of water molecules to the OH groups of wood cells. Accordingly, this significantly changed the WA values in the panels.

## 4. Conclusions

This experimental study showed that the properties of geopolymer-bonded plywood samples are significantly influenced by the various manufacturing parameters (lay-up times, compression ratio, veneer roughness, press temperature, and veneer layout). Plywood panels, produced with the lowest lay-up time (one minute), had the highest shear strength and bending properties. Increasing the compression ratio did not positively influence the shear strength of the geopolymer-bonded plywood. Although the bending strength of the samples improved by increasing the panel compression ratio, the panel density was also unfavourably increased. Increasing the veneer roughness had the best influence on improving the plywood properties. Plywood produced with rough veneers using gear wheels had a nearly 40% and 20% higher shear and bending strength, respectively, compared to that produced by the smooth veneers due to the improved entanglement of the wood-binder and the bonding quality. A press temperature of 140 °C is preferred to produce geopolymer-bonded plywood because it offers the best geopolymerisation process and binder features. Varying the veneer layouts also had an important role in determining the plywood properties and provides the opportunity to manufacture panels with different strength characteristics. As a recommendation, more research is needed to obtain comprehensive data on the effects of these processing parameters on the efficiency of plywood manufacturing, especially concerning the technical and economic aspects of these processes.

## Figures and Tables

**Table 1:** Different manufacturing parameters in the geopolymer-bonded plywood panels.

Variables	Lay-Up Time (min)	Compression Ratio of Panels (%)	Veneer Roughness	Press Temperature (°C)	Veneer Layout (Layers Degree) *
Lay-up time (min)	1	10	Medium	140	90-0-90
	5				
	10				
	15				
Compression ratio of panels (%)	1	5	Medium	140	90-0-90
		10			
		15			
		30			
Veneer roughness	1	5	Low	140	90-0-90
			Medium		
			High		
Press Temperature (°C)	1	5	High	120	90-0-90
				140	
				160	
Veneer layout (layers degrees) *	1	5	High	140	[1st] 90-0-90
					[2nd] 45-0-45
					[3rd] 45-90-45
					[4th] 22.5-45-67.5

* The position (angle) of the middle layers relative to the surface layers (veneer layouts).
